# Effects of Surface Modification on the Mechanical Properties of Flax/β-Polypropylene Composites

**DOI:** 10.3390/ma9050314

**Published:** 2016-04-27

**Authors:** Chang-Mou Wu, Wen-You Lai, Chen-Yu Wang

**Affiliations:** 1 Department of Materials Science and Engineering, National Taiwan University of Science and Technology, Taipei 10607, Taiwan; chris01_lay@hotmail.com.tw; 2 Department of Fiber and Composite Materials, Feng-Chia University, Taichung 40724, Taiwan; wcyben0822@gmail.com

**Keywords:** natural-fiber, composites, surface treatment, mechanical properties, beta-polypropylene

## Abstract

The effects of surface treatment of flax fibers featuring vinyltrimethoxy silane (VTMO) and maleic anhydride-polypropylene (MAPP) on the mechanical properties of flax/PP composites were investigated. α-polypropylene (α-PP) and β-polypropylene (β-PP) were used as matrices for measuring the mechanical properties of the flax fiber/polypropylene (flax/PP) composites. Flax/PP composites composed of double-covered uncommingled yarn (DCUY) were prepared using a film-stacking technique. The influence of surface treatment on the tensile, flexural, impact, and water uptake properties of Flax/PP composites were investigated. MAPP treatment was suitable for flax/PP composites in terms of superior tensile and impact properties. VTMO treatment showed superior flexural properties and less influence on the impact properties after moisture absorption.

## 1. Introduction

Research involving natural fiber-reinforced composites has grown in the academic and industrial sectors because of the high environmental friendliness, good mechanical properties, low energy consumption, cost efficiency, and easy end-of-life-cycle disposal of such materials, which contrasts with other available materials in this category [1,2,3,4,5,6,7,8,9,10,11]. Natural fibers such as flax, hemp, sisal, bamboo, and jute are alternatives to the use of glass fibers as reinforcement in polymer composites. Of these, flax is an attractive option because it is a cost-effective and low-density renewable raw material (approximately 1.4 to 1.5 g/cm^3^) with a highly specific strength and modulus [10]. Furthermore, flax fiber is significantly less abrasive than glass fiber. Flax composites have attracted attention to be considered as the next generation materials for structural application for infrastructure, the automotive industry, and consumer applications [12,13,14,15,16]. However, flax fibers have low thermal stability, high moisture uptake, and limited fiber length, and the properties of these plant fibers are strongly influenced by climate and location. Furthermore, the mechanical properties of flax fibers are also affected by manufacturing processes such as retting, scutching, bleaching, and spinning [17,18], which influence the final composite properties.

The primary advantages of using polypropylene (PP) as a matrix include its favorable properties, cost efficiency, and relatively low processing temperature requirements, which are essential because of the low thermal stability of flax fibers. The major limitations of using flax fibers to reinforce such matrices include the poor interfacial adhesion between polar hydrophilic fibers and nonpolar hydrophobic matrices, as well as difficulties in mixing because of poor wetting of fibers within the matrix. Weak bonding at the interface between the flax fibers and the polymer matrix is a common cause of the reduced mechanical strength of such composites. Furthermore, flax composites exhibit poor environmental and dimensional stability. Amorphous cellulose and hemicelluloses are primarily responsible for the high water uptake of flax fibers. Therefore, physical or chemical modification of flax fibers is necessary to improve the compatibility and adhesion between fibers and matrices. Various chemical treatments have shown improved moisture resistance in flax fibers and increased interfacial bonding with polymer matrices [1,2,3,19,20]. Specific alkali pretreatments are commonly used in natural fiber composites to transform cellulose I to cellulose II, which increases the molecular orientation, remove impurities, and leads to better surface roughness and fiber fibrillation [17,19,20,21,22,23]. Various chemical treatments such as maleic anhydride and maleic anhydride-PP (MAPP) [3,24,25,26,27,28], acetic anhydride [29], silane [2,3,30,31,32,33], and styrene [34] can react with the hydroxyl groups on the natural fiber surface. Those chemical treatments were not only used to modify the fiber surface, but also the PP matrix, to achieve better interfacial bonding and mechanical properties in composites. The mechanisms of the chemical treatments to improve their composite durability and mechanical properties were reported by Yan *et al.* [11].

Isotactic PP is one of the most common polymeric materials for natural fiber-reinforced composites. Commercial-grade PP crystallizes into the stable α-form with sporadic β-form crystalline structure formation. However, when special crystallization procedures are applied, or specific nucleators are added, the β-form can become a predominant crystalline form [35,36]. Recently, β-PP has attracted the interest of numerous scholars because it possesses some advantageous mechanical properties, such as high toughness, drawability, and low thermal deformation temperature compared to α-PP. We have reported surface modification of flax fibers by MAPP and vinyltrimethoxy silane (VTMO) to alter the interfacial bonding of PP resins [37]. However, no study on the mechanical properties of fiber-reinforced β-PP composites currently exists in the literature. This study investigates the effects of MAPP and VTMO treatments on the mechanical properties of flax/β-PP. The interfacial performances of flax/α-PP composites are also evaluated for comparison.

## 2. Results and Discussion

### 2.1. Tensile Properties

**Figure 1:**
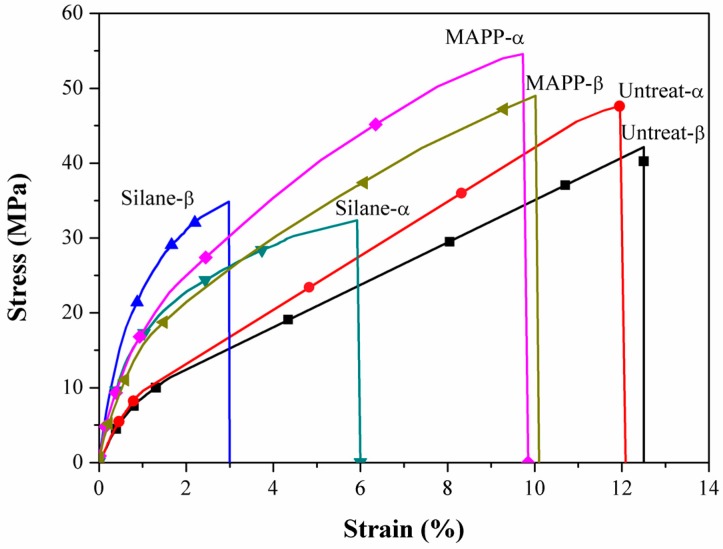
Typical tensile stress-strain curves of flax/PP composites.

**Figure 2:**
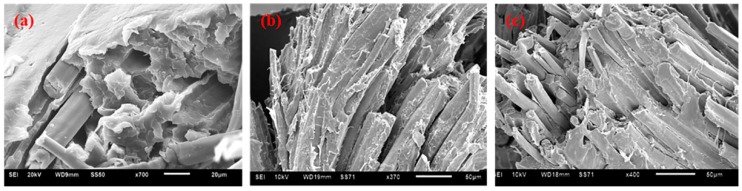
SEM images for the tensile damaged α-PP samples (**a**) untreated; (**b**) MAPP; and (**c**) VTMO.

**Figure 3:**
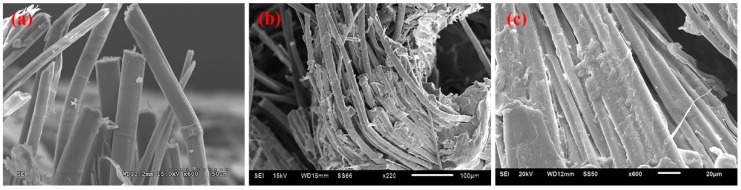
SEM images for the tensile damaged β-PP samples (**a**) untreated; (**b**) MAPP; and (**c**) VTMO.

**Figure 4:**
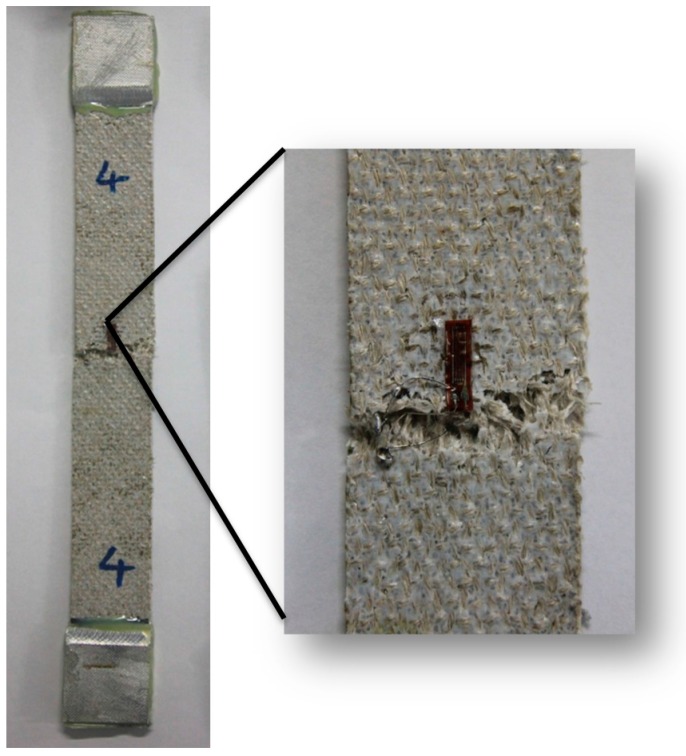
Typical tensile failure of flax/PP composites.

The tensile stress-strain curves of the flax/PP composites (Figure 1) show significant yielding and post-yield strain hardening. This nonlinear response should be attributed to an elasto-visco-plastic deformation of flax fiber, especially of the thickest cell wall (S2), since the alignment of the cellulosic micro-fibrils with the tensile axis led to the re-arrangement of the amorphous parts of the wall. The yield strength increased significantly in both MAPP and VTMO treated samples, indicating that surface treatment improved the interfacial bonding between flax and PP (α-PP and β-PP) [37]. The above findings were also confirmed through SEM analysis of α-PP (as shown in Figure 2) and β-PP (as shown in Figure 3) lamina, which are separated from the composites after tensile testing. The SEM micrographs as shown in Figure 2 and Figure 3 for the MAPP and VTMO samples corroborate the good interfacial bonding between the constituent materials. All of the MAPP- and VTMO-treated flax fiber bundles were coated with PP (α-PP or β-PP) resins. By contrast, the untreated α-PP and β-PP samples have no matrix on the flax fiber surface which demonstrates a poor interfacial bonding. Table 1 lists the summarized mechanical properties of the flax/PP composites, namely the tensile strength, tensile modulus, and elongation. MAPP treatment yielded the highest tensile strength, while VTMO exhibited the highest modulus. By contrast, VTMO showed the lowest elongation (6.0% for α-PP and 2.7% for β-PP) and the MAPP and untreated samples exhibited similar high values (10.7%–12.5%). This low elongation can be attributed to the crosslinking caused by VTMO treatment. Compared with the untreated flax/α-PP composites, MAPP treatment exhibited a higher tensile modulus (2.97 GPa) and tensile strength (53.1 MPa), representing increases of 90% and 14%, respectively. Similarly, MAPP treated flax/β-PP composites exhibited a higher tensile modulus (2.65 GPa) and tensile strength (48.8 MPa), representing increases of 60% and 14% respectively, compared to untreated samples. These results are consistent with those reported previously for oil palm empty fruit bunch/PP composites with MAPP and silane treatments [38]. Thus, in this study MAPP treatment is a viable method for use with flax/PP composites. Figure 4 shows a typical tensile damaged flax/PP sample. No difference was observed in the failure appearance for all treated and untreated flax/PP composites. The samples underwent break-apart failures, which involved yarn fracture, fiber pullout, and resin fracture. However, no shear failure and delamination was observed, confirming superior interfacial adhesion (as shown in Figure 2 and Figure 3).

### 2.2. Flexural Properties

**Figure 5:**
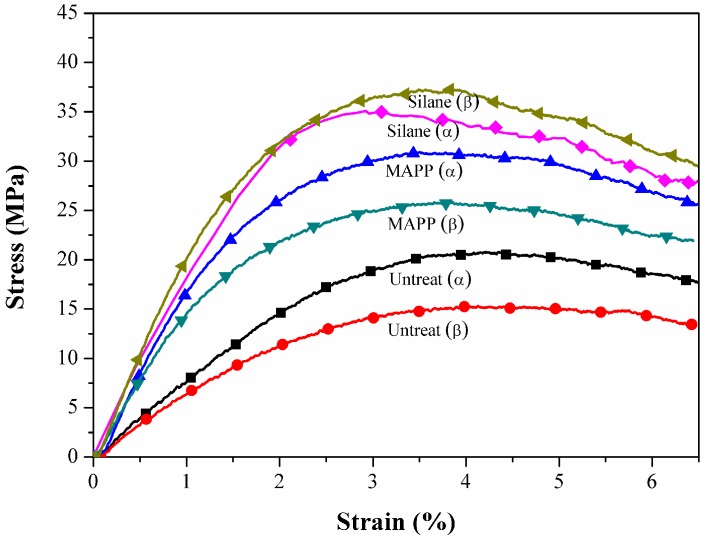
Typical flexural stress-strain curves of flax/PP composites.

**Figure 6:**
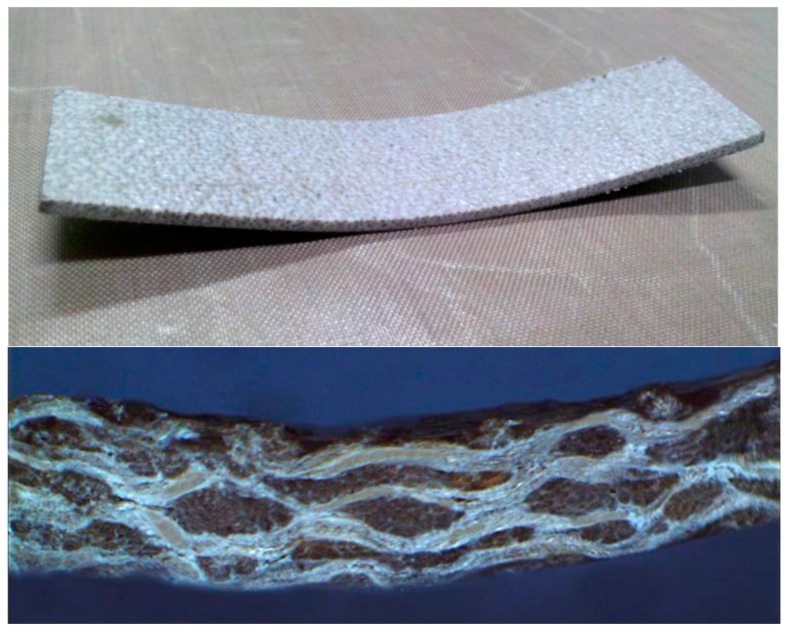
Typical flexural failure of flax/PP composites.

Figure 5 shows typical flexural stress-strain curves of flax/PP composites prepared using various surface treatments. The flax/PP composites did not collapse within the crosshead limit. It was attributed to the plastic behavior of the PP matrix (Figure 6). Stress whitening at the tensile side of the flexural specimen can be observed, indicating that microcracks may exist in the bend sample. The flax/β-PP samples treated using VTMO exhibited the optimal flexural properties; furthermore, the VTMO samples (Table 2) yielded the highest flexural modulus and strength at 2.19 GPa and 37.8 MPa, respectively. By contrast, the untreated composites exhibited the lowest flexural modulus (0.59 GPa) and strength (15.5 MPa) values, decreases of 73% and 58% compared with the VTMO composites, respectively. It is worth noting that MAPP is also an effective method for improving the flexural properties of the flax/PP composites. Trends similar to those in the tensile properties were observed in the flexural properties of MAPP samples. MAPP-treated flax/β-PP composites exhibited a higher flexural modulus (1.13 GPa) and tensile strength (25.8 MPa), increases of 92% and 66% compared to untreated samples, respectively. The added MAPP used in this study bridges the flax fibers and PP matrix through chemical covalent bonding and compatibility improvements.

### 2.3. Impact Properties

Table 2 lists the notched Izod impact energy of the flax/PP composites. The impact energy of the flax/PP samples prepared using various surface treatments ranged from 263 to 466 J/m. The VTMO treated flax/PP composites yielded the lowest impact energy, which can be attributed to the high crosslink PP structure in the vicinity of flax fiber. The VTMO samples underwent break-apart failures and exhibited the lowest impact energy levels. By contrast, the impacted MAPP composites using α-PP and β-PP did not break apart, but exhibited tensile and compressive failures on two sides of the impacted specimen. The tensile sides of the MAPP samples exhibited severe fiber pullout and breakage. By contrast, the compressive sides exhibited severe compressive shearing failures accompanied by fiber breakage (kinks and buckles), crushed matrices, and delamination. Compared with the untreated samples, MAPP samples led to higher impact energies in α-PP composites (466 J/m) and β-PP (437 J/m), representing increases of 42% and 19%, respectively.

### 2.4. Effects of Water Uptake on the Flexural and Impact Properties of Flax/PP Composites

**Figure 7:**
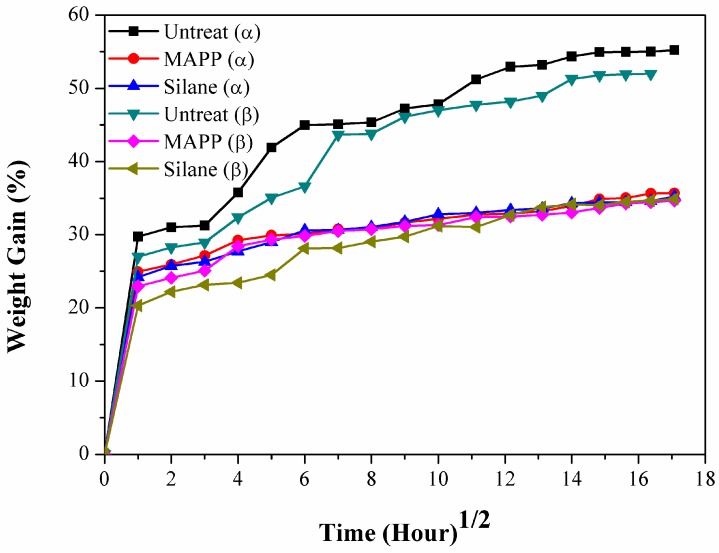
Water uptake of flax/PP composites.

Figure 7 shows the water uptake with immersion time of flax/PP composites in boiling water. Surface treatment greatly reduced the water uptake of the flax/PP composites, but the water uptake increased with immersion time. The increase in weight was not consistent with respect to the immersion time. At the base of the curve, the weight increased sharply, which demonstrates rapid moisture penetration into the composite materials. This phenomenon was attributed to the permeability of the material and capillary action, where it becomes active as water penetrates into the interface through the voids induced by swelling of the flax fibers. The rate of water absorption slowed after one day of immersion, reaching saturation. For untreated α-PP and β-PP samples, the water uptake at saturation is 56% and 52%, respectively. However, no difference in the water uptake value (35%) is observed for all surface-treated samples.

**Figure 8:**
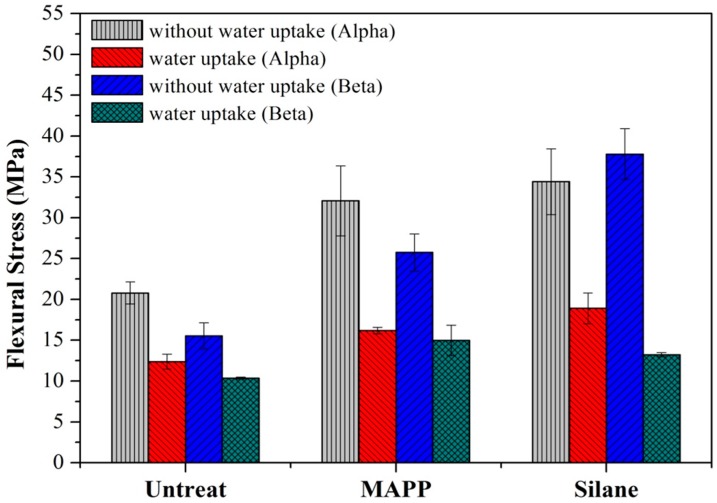
Effects of water uptake on the flexural properties of flax/PP composites.

**Figure 9:**
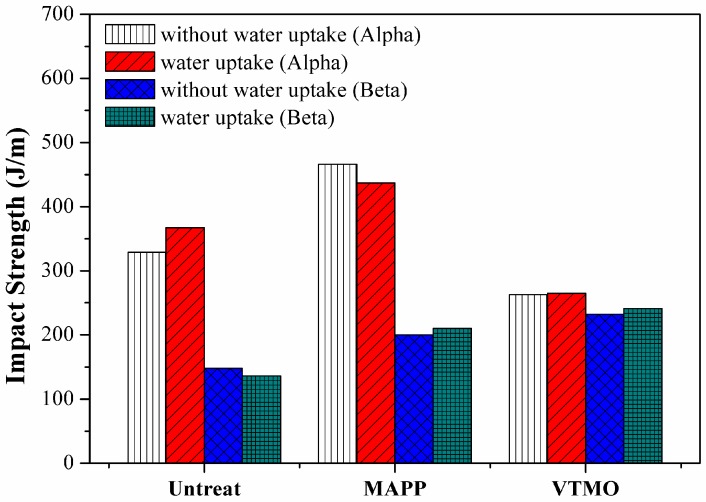
Effects of water uptake on the impact properties of flax/PP composites.

Figure 8 shows the effects of water uptake on the flexural strength of flax/PP composites. The flexural strength decreased significantly after water absorption for all flax/PP samples. Though the flexural strengths for the MAPP and VTMO samples were higher than the untreated samples, these differences are smaller compared to the dry samples. The effects of water uptake on the impact energy of flax/PP composites are shown in Figure 9, showing trends similar to those of the flexural properties. The impact energy decreased significantly after water absorption, with the impact energy for the VTMO samples showing the highest values. This indicates that interfacial bonding is effective in hindering moisture penetration. Softening of the crosslink PP in the vicinity of the flax fiber may also contribute to the decreased impact energy. 

## 3. Experimental

### 3.1. Materials

Flax fiber bundles were obtained using retting processes, which involved the biological movement of bacteria in an aqueous medium where pectin and wax were removed. Flax yarns with a linear mass density of 27 tex were purchased from New Fiber Textile Corporation, New Taipei City, Taiwan. α-PP and β-PP draw texture yarns (DTY) with a linear mass density of 50 tex/96 filaments were fabricated by Tri Ocean Textile Corporation, Taipei, Taiwan. Commercial-grade isotactic PP (2123, Formosa Plastic Corporation, Taipei, Taiwan) was used as a base material throughout the study; the material has a melt flow index of 25 g/10 min (2.16 kg, 230 °C) and a density of 0.90 g/cm^3^. To prepare β-PP, a specific β-nucleating agent (NAB 83, GCH technology Int., Guangzhou, China) and the original α-PP were mechanically mixed and subsequently processed into pellets using a twin-screw extruder. The nucleating agent was added at 0.15 wt %, which is the concentration at which the β-form content reaches its saturation level. The tensile properties for various surface-treated flax yarns and PP yarns were listed in Table 3. The tenacity values for α-PP and β-PP DTYs are 39.64 cN/tex (7.13 MPa) and 30.94 cN/tex (5.57 MPa), respectively. NaOH was used for the flax fiber surface pretreatment. VTMO silane (VTMO, Shin-Etsu, Taipei, Taiwan), MAPP copolymer (MAPP P613, Dupont, Wilmington, DE, USA), and dicumyl peroxide (DCP, ECHO Chemical, Miaoli, Taiwan) were used as coupling agents. The tenacity values for the untreated, MAPP and VTMO treated flax yarn are 230.57 cN/tex, 274.16 cN/tex, and 300.23 cN/tex, respectively.

### 3.2. Fiber Surface Treatment

The flax surface treatments have been described previously [37]. Flax fibers were alkalized prior to subsequent treatment. The flax fibers were then treated by MAPP, which bonded by esterification. VTMO treatment initiated the grafting mechanism by the decomposed dicumyl peroxide radicals. Both treatments are effective for composite interfacial adhesion.

### 3.3. Sample Preparation

**Figure 10:**
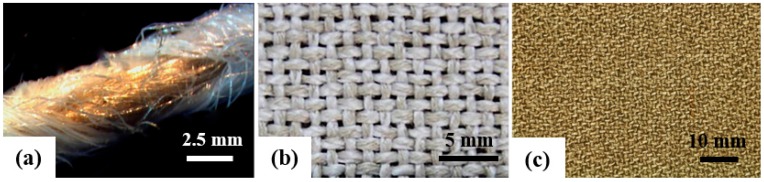
Photo images of (**a**) the double covered uncommingled yarn; (**b**) the flax/PP preform; and (**c**) the flax/PP composites.

Flax and PP yarn designed at a 45/55 volume fraction were used to prepare the double-covered uncommingled yarns (DCUYs; Figure 10a) using a hollow-spindle spinning machine [39,40,41]. The flax yarn was used as the reinforcing core yarn, and PP multifilament yarn was used as the wrapping material, forming linear co-wrap spinning yarn preforms. Yarn stability primarily depends on the binding yarn and twist introduced during spinning. The PP filaments served as carriers for the flax yarn during processing and became the polymer matrix in the final composites, facilitating impregnation and preventing damage to the reinforcing flax yarn. To ensure that the distribution of the fiber and thermoplastic resin in the preforms was even and that the fiber content was appropriate (45 wt %), the spinning parameters (hollow spindle twist: 776 turns/m and hollow spindle rotational speed: 5554 rpm) were optimized.

DCUY was used as a feed material for the production of plain woven structure preforms. The density of wrap and weft was 8 yarns/cm and 6 yarns/cm, respectively. Figure 10a shows the surface of the co-wrap spinning yarn, the wrapped angle between PP multifilaments, and the reinforcing flax core yarn axis. By changing the co-wrap spinning parameters, different reinforced flax fiber contents could be achieved. The co-wrap spinning yarns (acting as warp and weft yarns) were then woven on a shuttle loom. Figure 10b shows the appearances of the flax/PP perform.

This study presents a modified film-stacking technique used to produce high-quality, impregnated, and void-free (<1%) flax/PP composites. Single laminae were prepared by hot pressing the preform at 180 °C for 1 min at a pressure of 50 kg/cm^2^, and then quenching the samples in water. Flax/PP laminates (Figure 10c) were prepared by stacking four layers of laminae at 200 °C for 3 min at a pressure of 100 kg/cm^2^ followed by slow cooling to room temperature (RT) and demolding. The fiber volume fractions of the flax/PP composites were approximately 44%.

### 3.4. Mechanical Tests

A universal testing machine (AG-100kNX, Shimadzu, Tokyo, Japan) was used to conduct the tensile tests and three-point bending flexural tests at RT according to the ASTM D638 (type I), D3039, and D790 standards, respectively. The dimensions of the tensile specimens cut from the prepared flax/PP samples were 250 × 25 × 2 mm^3^, and an area of 50 × 25 mm^2^ was clamped at each end, leaving a gauge length of 150 mm. Aluminum tabs were glued to the ends of the specimens to facilitate gripping, and the grip pressure was hydraulically controlled. The testing crosshead speeds were 5 mm/min for the tensile test. The specimen size for the three-point bending test was 100 × 25.4 × 2 mm^3^. A span length of 64 mm ensured that the span-to-depth ratio was 32, and crosshead speeds of 3.4 mm/min were adopted. The Izod impact test was performed at RT according to the ASTM D256 standard by using a pendulum impact tester (CPI, Atlas electric devices, Chicago, IL, USA) at an impact energy of 5.4 J. The impact velocity was 3.4 m/s. The dimensions of the Izod impact specimen were 63.5 × 12.7 × 2 mm^3^, and the specimens contained a 2.7 mm (±0.2 mm) deep notch. The reported mechanical properties represent the average value of at least five readings. Damaged specimens were inspected using stereo microscopy (S422L, Microtech, Taipei, Taiwan) and scanning electron microscopy (SEM; S3000, Hitachi, Tokyo, Japan) to characterize their failure modes. Prior to SEM observations, the samples were mounted on aluminum stubs and sputter-coated with a thin layer of gold to prevent electrical charging. SEM micrographs were captured at a 10 kV acceleration voltage at various magnification levels.

## 4. Conclusions

This study investigated the effects of MAPP and VTMO treatments on the mechanical properties of flax/β-pp composites. The influence of surface treatment on the tensile, flexural, impact, and water uptake properties of flax/PP composites were investigated. According to the experimental results, surface-treated flax/PP composites exhibited markedly improved tensile, flexural, and impact properties. MAPP treatment successfully improved the tensile and impact properties of flax/PP composites, as it bridges the flax fibers and the PP matrices through chemical covalent bond formation and compatibility improvements. VTMO treatment led to superior flexural properties, as VTMO treatment formed crosslink PP structures near the flax fiber, which hindered the moisture absorption and, thus, exhibited less influence on the impact properties.

## Figures and Tables

**Table 1:** Tensile properties of flax/PP composites.

Resin	α-PP			β-PP		
Treatment	Untreated	MAPP	VTMO	Untreated	MAPP	VTMO
Strength (MPa)	46.4 ± 2.4	53.1 ± 2.8	31.8 ± 3.7	42.8 ± 0.6	48.8 ± 2.8	34.5 ± 4.0
Strain (%)	12.1 ± 1.1	10.7 ± 1.6	6.0 ± 0.7	12.5 ± 0.9	11.1 ± 0.2	2.7 ± 0.3
Modulus (GPa)	1.56 ± 0.23	2.97 ± 0.62	3.01 ± 0.44	1.66 ± 0.22	2.65 ± 0.15	3.97 ± 0.15

**Table 2:** Flexural and impact properties of flax/PP composites.

Resin	α-PP			β-PP		
Treatment	Untreated	MAPP	VTMO	Untreated	MAPP	VTMO
Strength (MPa)	20.8 ± 1.4	32.1 ± 4.3	34.4 ± 4.0	15.5 ± 1.6	25.8 ± 2.3	37.8 ± 3.1
Modulus (GPa)	0.81 ± 0.08	1.73 ± 0.19	1.96 ± 0.23	0.59 ± 0.07	1.13 ± 0.17	2.19 ± 0.13
Impact energy (J/m)	329 ± 21	466 ± 3	263 ± 19	367 ± 18	437 ± 19	265 ± 11

**Table 3:** Tensile properties of various surface treated flax yarns and PP yarns.

Sample	Strength (cN/tex)	Strain (%)
Flax (Untreated)	230.57	0.86
Flax (VTMO)	300.23	1.25
Flax (MAPP)	274.16	1.12
α-PP	39.64	29.63
β-PP	30.94	32.04
